# Effectiveness and safety of antifibrinolytic agents in off-pump coronary artery bypass grafting: a systematic review and meta-analysis

**DOI:** 10.1016/j.bjane.2026.844731

**Published:** 2026-01-24

**Authors:** João Lucas W.C. Marchesani, Matheus H. Leite e Silva, Matheus S. Thomaz, Davi B. Wolff, Emerson C.L. Almeida, Michelle D.S.S. Costa

**Affiliations:** a Faculdade de Medicina da Universidade Federal de Minas Gerais, Belo Horizonte, MG, Brazil; b Faculdade de Medicina da Universidade Federal de Minas Gerais, Department of Surgery, Belo Horizonte, MG, Brazil

**Keywords:** Antifibrinolytic agents, Aprotinin, Myocardial revascularization, Tranexamic acid

## Abstract

**Background:**

Coronary Artery Bypass Grafting (CABG) is the most widely used cardiac intervention and can be accomplished without an extracorporeal circulation off-pump. The benefits of antifibrinolytics in off-pump CABG have yet to be demonstrated.

**Methods:**

Randomized Controlled Trials (RCTs) and observational studies comparing the use of antifibrinolytic agents (tranexamic acid, aprotinin, and epsilon-aminocaproic) versus controls in patients undergoing off-pump CABG were searched in the PubMed, Embase, and Cochrane databases. Outcomes included thromboembolic events, in-hospital mortality, overall mortality, bleeding, Intensive Care Unit (ICU) length of stay, and blood product transfusions. Meta-analyses were conducted using the Inverse Variance method under a random-effects model, with p < 0.05 considered statistically significant.

**Results:**

Of the 23,149 patients in 20 RCTs and seven observational studies, 51% received antifibrinolytic agents (tranexamic acid or aprotinin). Observational and randomized designs were analyzed separately in primary subgroup analyses. Significant reduction was found for overall mortality (RR = 0.72; [95% CI 0.54–0.95]) in the RCT subgroup. Red-blood-cell transfusion requirements were reduced (RR = 0.65; [95% CI 0.53, 0.78]) as well as Platelets (RR = 0.59; [95% CI 0.41, 0.84]) and fresh-frozen-plasma (RR = 0.39; [95% CI 0.36, 0.42]) transfusion requirement. The RCT subgroup showed a reduction in thromboembolic events (RR = 0.55; [95% CI 0.39, 0.79]).

**Conclusions:**

In *off-pump* CABG, antifibrinolytics reduced the need for blood transfusion while reducing thromboembolic events and overall mortality in RCT subgroups, while pooled analyses combining RCTs and observational studies did not demonstrate significant reductions.

**Prospero Link:**

https://www.crd.york.ac.uk/PROSPERO/view/CRD42024545640

## Introduction

Coronary Artery Bypass Grafting (CABG) is the most commonly performed cardiac procedure worldwide, with the possibility of being carried out with the use of Cardiopulmonary Bypass (CPB), referred to as “on-pump”, or without it, referred to as “off-pump”.^1^ On-pump CABG allows greater stabilization of the anastomosis site, enhanced myocardial protection achieved through cardioplegia, and improved handling of the heart by surgeons. However, blood contact with the CPB circuit can activate clotting, exhaust coagulation factors, and cause platelet dysfunction and excessive fibrinolysis.^2^ Off-pump CABG is an alternative for patients undergoing CPB and aortic clamping, particularly those with heavily calcified or atheromatous aortas, or those at a high risk of dissection or embolization. While avoiding CPB-related risks, including blood exposure, hypothermia, acidosis, and tissue trauma, cardiac surgeries inherently carry a high risk of perioperative bleeding.^3^ This raises specific concerns regarding hemostasis, as reducing blood loss is crucial for preventing worse patient outcomes.^2^

In this context, the use of antifibrinolytic agents to improve hemostasis is a viable approach for both on- and off-pump cardiac surgery. The clinical relevance of these agents is centered on mitigating perioperative bleeding and consequently reducing the need for allogeneic blood transfusions, which are independent risk factors for postoperative morbidity and mortality. Although off-pump CABG prevents systemic coagulopathy induced by cardiopulmonary bypass, extensive surgical trauma can trigger major fibrinolysis. Acknowledging this risk, prophylactic antifibrinolytic administration has become a cornerstone of modern blood conservation strategies, a practice strongly advocated by major clinical practice guidelines.^4^^,^^5^

Antifibrinolytic agents act primarily by inhibiting plasminogen activation and plasmin activity but differ in specificity and potency. Tranexamic Acid (TXA) and Epsilon-Aminocaproic Acid (EACA) are synthetic lysine analogs that competitively block plasminogen binding to fibrin, thereby preventing its conversion to plasmin and reducing fibrin clot degradation. In contrast, aprotinin is a serine protease inhibitor that directly blocks plasmin, kallikrein, and trypsin. Thus, while TXA and EACA primarily interfere with plasminogen activation, aprotinin provides more potent and pleiotropic inhibition of fibrinolysis.

The use of antifibrinolytics in on-pump CABG has been well established.^6^ However, it remains uncertain whether off-pump CABG offers significant clinical benefits. In CABG, the absence of cardiopulmonary bypass reduces blood contact with extracorporeal circuits, leading to lower inflammatory activation, attenuated fibrinolysis, and decreased consumption of coagulation factors compared with on-pump surgery. Consequently, bleeding risk and transfusion requirements are reduced. Under these conditions, the effect of lysine analogs such as TXA and EACA may be less pronounced, as their antifibrinolytic action is most evident in settings of hyperfibrinolysis. Aprotinin, with its broader antifibrinolytic profile, effectively reduces the need for blood product transfusion; however, its additional effects have limited its use due to safety concerns. Previous meta-analyses have demonstrated the ability of these drugs to reduce perioperative bleeding and, consequently, the number of transfusions.^7^ However, currently available studies and observational studies were excluded from the analyses. This systematic review and meta-analysis aimed to provide a comprehensive and current evaluation of antifibrinolytic use in off-pump CABG. It incorporates more Randomized Controlled Trials (RCTs) and includes observational studies that contribute to a substantial number of patients, facilitating comparisons with smaller RCTs, as well as including studies published after the most recent meta-analysis on the topic. In addition, compared with the most recently published meta-analysis, the present study evaluated mortality and thromboembolic events as independent outcomes, evaluated each thromboembolic outcome individually, and further divided mortality into in-hospital and overall mortality subgroups, thereby enabling more accurate interpretation of the data.^7^

## Methods

This study was conducted and reported based on the Preferred Reporting Items for Systematic Reviews and Meta-analyses (PRISMA) and the Cochrane Guidelines.^8^ The protocol was registered with the International Prospective Register of Systematic Reviews (PROSPERO; identifier: CRD42024545640) on May 24, 2024.

### Eligibility criteria

Inclusion and exclusion criteria were defined according to the prospectively defined PICOTT framework: P – Adult patients undergoing off-pump CABG; I – Anti-fibrinolytic therapy; C – Placebo or no intervention; O – Overall mortality, thromboembolic events, ICU length-of-stay, blood transfusion requirements, and intraoperative blood loss; T – Randomized controlled trials and observational studies; and T – No time constraints in this study.

The inclusion in this systematic review and meta-analysis was restricted to studies with the following eligibility criteria: 1) RCTs or non-RCTs; 2) Patients who underwent off-pump CABG; and 3) Comparison of antifibrinolytics (TXA, EACA, or aprotinin) with placebo or no intervention. Studies were included only if they had at least one previously determined outcome, and no limits were established for the follow-up duration.

The following study designs were excluded: 1) Reviews; 2) Meta-analyses; 3) Guidelines; and 4) Communications. Additionally, studies were excluded if they involved population overlap, lacked a control group, did not consider antifibrinolytic use, were available only as abstracts, or assessed mediastinal antifibrinolytic application.

### Search strategy and data extraction

We systematically searched PubMed, Embase, and Cochrane for studies published up to July 18, 2024, without language restriction, with the following search strategy: (“off pump” OR “off-pump” OR “op-cab” OR opcab) AND (“Tranexamic acid” OR Antifibrinolytic OR “Antifibrinolytics” OR cyklokapron OR transamin OR amcha OR TXA OR “epsilon-aminocaproic acid” OR “epsilon aminocaproic acid” OR aprotinin). The complete search strategy for each database is provided in the Supplementary Material. Two authors (JLW and MS) independently extracted the data using predefined search criteria and quality assessments. Disagreements between the two reviewers were resolved through discussion with a third reviewer (DW). After removing duplicates, all the studies were screened based on their titles and abstracts. The remaining articles were then thoroughly read. A Zotero Tool was manually used to perform this process. If the number of patients in the outcomes was expressed as a percentage and the conversion for absolute numbers resulted in a decimal number, the number was approximated to the closest integer.^9^, ^10^, ^11^, ^12^

### Endpoints and subgroup analyses

Outcomes included overall mortality, intrahospital mortality, thromboembolic events, time spent in the Intensive Care Unit (ICU), platelet transfusion, plasma transfusion, red blood cell transfusion, and intraoperative blood loss. Transfusion was quantified by the number of patients who received blood components. In some cases, RCTs and non-RCTs were analyzed independently to better elucidate the outcomes.

In addition, the impact of different anti-fibrinolytics was investigated by conducting a subgroup analysis, as shown in the. Sensitivity analyses were conducted by excluding high-impact studies. If an outcome of interest was described as a secondary outcome in the original trial, it still was included in the analysis to avoid selection bias. Studies that did not report outcome data were excluded from meta-analysis. No multiple imputation methods were applied to handle missing data.

In this review, overall mortality, intrahospital mortality and thromboembolic events were considered primary endpoints, whereas ICU length-of-stay, blood transfusion requirements, and intraoperative blood loss were considered secondary outcomes.

### Quality assessment

The risk of bias was evaluated in randomized studies using the second version of the Cochrane Risk-of-Bias assessment tool (RoB2), whereas non-randomized studies were assessed using the Risk of Bias in Non-randomized Studies of Interventions Tool (ROBINS-I) (Fig. S15).^13^^,^^14^ Two independent authors (JLW and EC) independently completed the risk-of-bias assessment. Disagreements were resolved through discussions with a third author (MS). The final figure was generated using robvis, a visualization tool designed for risk of bias assessment in systematic reviews.^15^ Publication bias was investigated using Egger’s tests and funnel plot analyses of effect measures in relation to study weights, which are available in the Supplementary Appendix.

### Statistical analysis

Random-effects inverse variance meta-analyses were conducted with restricted maximum likelihood estimates of tau^2^ and Hartung-Knapp confidence intervals. As between-study heterogeneity was expected, the individual true effect for each study could differ from the overall true effect; therefore, random-effects modeling was preferred. Heterogeneity was assessed using the *Q* test (α = 0.10) and described by the I^2^ statistic. The thresholds of 25%, 50% and 75% were used to define low, moderate and high heterogeneity, respectively. Estimates were reported as Relative Risks (RR) for dichotomous outcomes and Mean Differences (MD) for discrete variables with 95% Confidence Intervals. Subgroup analyses moderated by study design were conducted, and differences were tested by standard χ^2^ tests.

A standard continuity correction (addition of 0.5 to zero-value cells on both arms) was applied to the outcomes of interest to prevent null denominator analysis. Trials that did not report specific analyzed outcomes were excluded from the respective meta-analyses to which they could not contribute data. Analyses were performed using *R* version 4.3.3, employing the meta, ggplot2, dplyr, robvis packages and Review Manager version 5.4.^16^

### Certainty of evidence

The Grading of Recommendations Assessment, Development, and Evaluation (GRADE) system was used to assess the level of certainty of the evidence. This system consists of five domains: risk of bias, inconsistency, indirectness, imprecision, and publication bias. The overall quality was classified as high, moderate, low, or very low. The quality of all outcomes was assessed by two independent reviewers (JLW and MS), and any disagreements were resolved through discussion among all authors.

## Results

### Study selection and baseline characteristics

**Figure 1:**
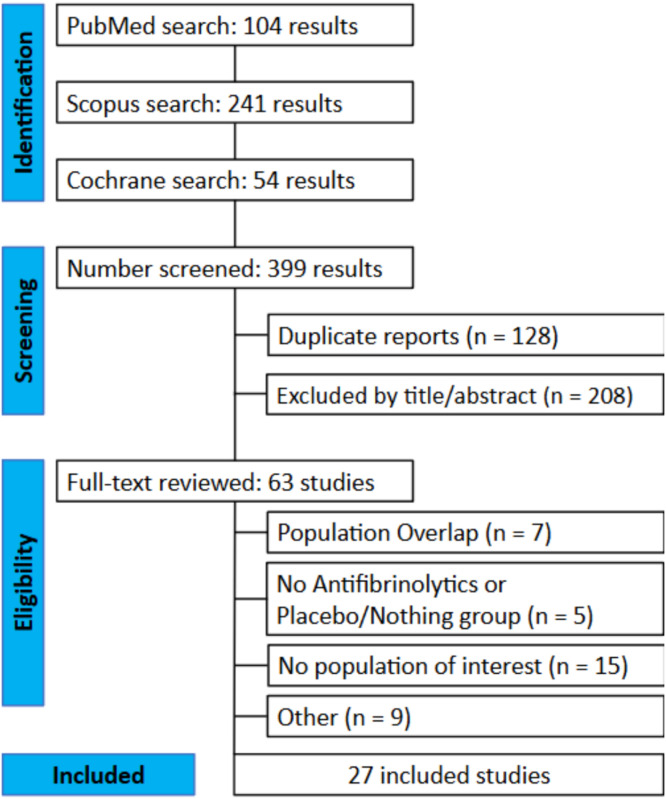
PRISMA flow diagram of study screening and selection. Study flow diagram.

The initial search yielded 399 results. After removing 128 duplicate records, 208 studies were excluded from the title/abstract analysis. Thus, 63 studies remained and were full-text reviewed based on the inclusion criteria (Fig. 1). Of these, 27 studies were included, including 23,149 patients from 20 RTCs and 7 non-randomized cohorts. A total of 11,899 (51%) patients received antifibrinolytics, and 11,250 (49%) received placebo or no intervention. In the antifibrinolytic group, 11,208 (94%) patients received TXA and 691 (6%) received aprotinin. The dose regimen presented significant variability among studies, but in the vast majority, it consisted of a bolus at the beginning of surgery, followed by an infusion until the end of surgery. Due to the specific type of patient with highly developed coronary disease that undergoes off-pump CABG, the populations in the different studies did not show a significant difference. Other characteristics, such as the type of control group, presence of a cell saver, operation time, and number of grafts, are shown in Table 1. Studies including EACA compared it with other antifibrinolytics, and no trials assessing EACA versus placebo were available. Therefore, this meta-analysis was restricted to TXA and/or aprotinin versus placebo.

**Table 1:** Baseline characteristics of included studies.

Study	Design	AF	Patients AF/PB	Control Group	Cell Saver	Male, % AF/PB	Age^†^, y AF/PB	BMI AF/PB	Operation Time	Preoperative Antiplatelet Agents AF/PB	Number of Grafts AF/PB	Ejection Fraction AF/PB
Ahn, 2012	RCT	TXA	38/38	Placebo	For all patients	60/47	69 ± 7 / 67 ± 7	NA	250 ± 47 / 246 ± 51	All agents interrupted 5 days before surgery	3.1 ± 0.6	60 ± 13 / 64 ± 10
Bert, 2008	RCT	Aprotinin	25/25	No AF	NA	80/80	65.7 ± 10.2 / 67.8 ± 10	NA	242.2 ± 51.5 / 260.9 ± 53.5	NA	NA	NA
Bittner, 2008	Non-RCT	Aprotinin	391/370	No AF	For all patients	81.1%	68.5 ± 9.3 / 67.8 ± 10	27.6 /27.5	163 ± 43 / 168 ± 51	337/320 (aspirin)	NA	NA
Casati, 2001	RCT	TXA	20/20	Placebo	NA	85/75	64 ± 13 / 62 ± 11	NA	NA	6/8 (aspirin)	NA	42 ± 14 / 45 ± 12
Casati, 2004	RCT	TXA	26/25	Placebo	Not for all	76/84	64 ± 12 / 61 ± 11	NA	NA	6/7 (aspirin)	NA	43 ± 14 / 46 ± 13
Chakravarthy, 2012	RCT	TXA	50/48	No AF	NA	78/81	NA	NA	300 ± 0.5 / 300 ± 0.5	All agents interrupted 7 days before surgery	7/6	NA
Desai, 2009	RCT	Aprotinin	37/38	Placebo	For all patients	NA	NA	NA	NA	Aspirin for all patients	NA	NA
Durand, 2006	Non-RCT	Aprotinin	40/40	No AF	NA	NA	67 ± 12 / 66 ± 12	NA	193 ± 49 / 156 ± 43	Aspirin for all patients	2.6 ± 0.7 / 2 ± 0.8	58 ± 15 / 55 ± 20
Englberger, 2002	RCT	Aprotinin	22/25	Placebo	NA	72/76	63.9 ± 10.8 / 66.4 ± 9	74.6 ± 14.1 / 77.1 ± 8.6	176 ± 40 / 177 ± 56	18/23 (aspirin)	2.9 ± 1 / 2.8 ± 1.2	65 ± 13 / 60 ± 13
Grant, 2008	RCT	Aprotinin	59/61	Placebo	For all patients	NA	NA	NA	NA	Aspirin	3.0 ± 0.8 / 2.8 ± 0.9	NA
Hosseini, 2014	RCT	TXA	35/36	Placebo	NA	NA	NA	NA	NA	NA	NA	NA
Hulde, 2019	Non-RCT	TXA	2249/2249	No AF	NA	81/79	67.2 ± 9.9 / 67.5 ± 9.6	28.2 ± 4.4 / 28.1 ± 4.4	NA	1696/1655 (aspirin)	2.9 ± 0.8 / 2.8 ± 0.8	56.5 ± 11 / 56.7 ± 11.3
Khadanga, 2020	Non-RCT	TXA	30/30	No AF	NA	NA	57.5 ± 7.8 / 57.9 ± 7.6	NA	NA	Aspirin interrupted on the day of surgery	NA	50.0 ± 7.8 / 50.2 ± 6.9
Kim, 2004	RCT	Aprotinin	15/15	Placebo	NA	46/60	66 ± 7.4 / 59.7 ± 6.8	NA	320 ± 64.3 / 345 ± 50.5	NA	2.7 ± 0.6 / 2.6 ± 0.4	57.2 ± 10.6 / 52.7 ± 11.1
Mehr-Aein, 2007	RCT	TXA	33/33	Placebo	No	36/45	44 ± 10 / 45 ± 10	23.4 ± 2.6 / 23.4 ± 3.3	NA	Suspended 7 days before operation	2.4 ± 0.3 / 2.3 ± 0.7	45 ± 8 / 40 ± 10
Mouton, 2008	Non-RCT	TXA	2140/1532	No AF	NA	80/81	65.3 ± 9.1 / 64.1 ± 9.6	NA	NA	NA	NA	NA
Murphy, 2006	RCT	TXA	50/50	Placebo	For all patients	74/84	64.9 ± 7 / 65.8 ± 8.7	27.3 ± 4.35 / 28.6 ± 4.49	210/240	5 patients treated with aspirin or heparin	3/3	NA
Nurözler et al; 2008	RCT	Aprotinin	25/26	Placebo	NA	76/69	63.1 ± 8.8 / 64.6 ± 6.7	28.1 ± 4.1 / 26.9 ± 3.7	NA	6/8 (aspirin)	NA	43.8 ± 6.2 / 42.3 ± 5.3
Poston et al., 2006	RCT	Aprotinin	29/31	Placebo	For all patients	NA	NA	NA	NA	All patients received aspirin	3.1 ± 0.3 / 3.3 ± 0.4	NA
Taghaddomi, 2008	RCT	TXA	50/50	Placebo	NA	76/68	54.7 ± 10.9 / 60.3 ± 10.2	NA	176.5 ± 44.7 / 174.4 ± 32.6	88/92 (aspirin)	3.7/3.8	52.7 ± 11.1 / 54.1 ± 9
Vanek et al., 2005	RCT	TXA	61/30	Placebo	NA	59/73	67.3/68.9	NA	141.7/152.1	Aspirin suspended 5 days before surgery	1.8/1.8	NA
Wang et al., 2022	Non-RCT	TXA	6184/6184	Placebo	NA	77/77	61.5 ± 8.7 / 61.6 ± 8.8	25.7 ± 3.1 / 25.7 ± 3.0	NA	NA	NA	NA
Vijay at al., 2023	RCT	TXA	30/30	Placebo	NA	66/60	NA	NA	NA	NA	NA	NA
Wei et al., 2006	RCT	Aprotinin	36/40	Placebo	NA	77/80	61.4 ± 7.5 / 60.7 ± 8	NA	195 ± 34.3 / 203.4 ± 29.4	Aspirin suspended 5‒7 days before surgery	3 ± 0.8 / 2.6 ± 0.6	63.5 ± 8.9 / 62.40 ± 9.4
Wei et al., 2006	RCT	TXA	36/40	Placebo	NA	77/80	62.8 ± 7.9 / 60.7 ± 8	NA	191.3 ± 47.1 / 203.4 ±2 9.4	Aspirin suspended 5‒7 days before surgery	2.8 ± 0.6 / 2.6 ± 0.6	61 ± 8.9 / 62.4 ± 9.4
Weingarten, 2021	Non-RCT	TXA	176/172	Placebo	NA	46/73	66.6 ± 9.7 / 65.6 ± 10.4	29.3/27.8	338 ± 66.5 / 326 ± 64.7	NA	NA	NA
Yang et al., 2003	RCT	Aprotinin	NA	Placebo	NA	NA	NA	NA	NA	NA	NA	NA

AF, Antifibrinolytics; PB, Placebo; BMI, Body Mass Index; RCT, Randomized Controlled Trial; Non-RCT, Observational Studies; NA, Not Available.

### Pooled analyses

**Figure 2:**
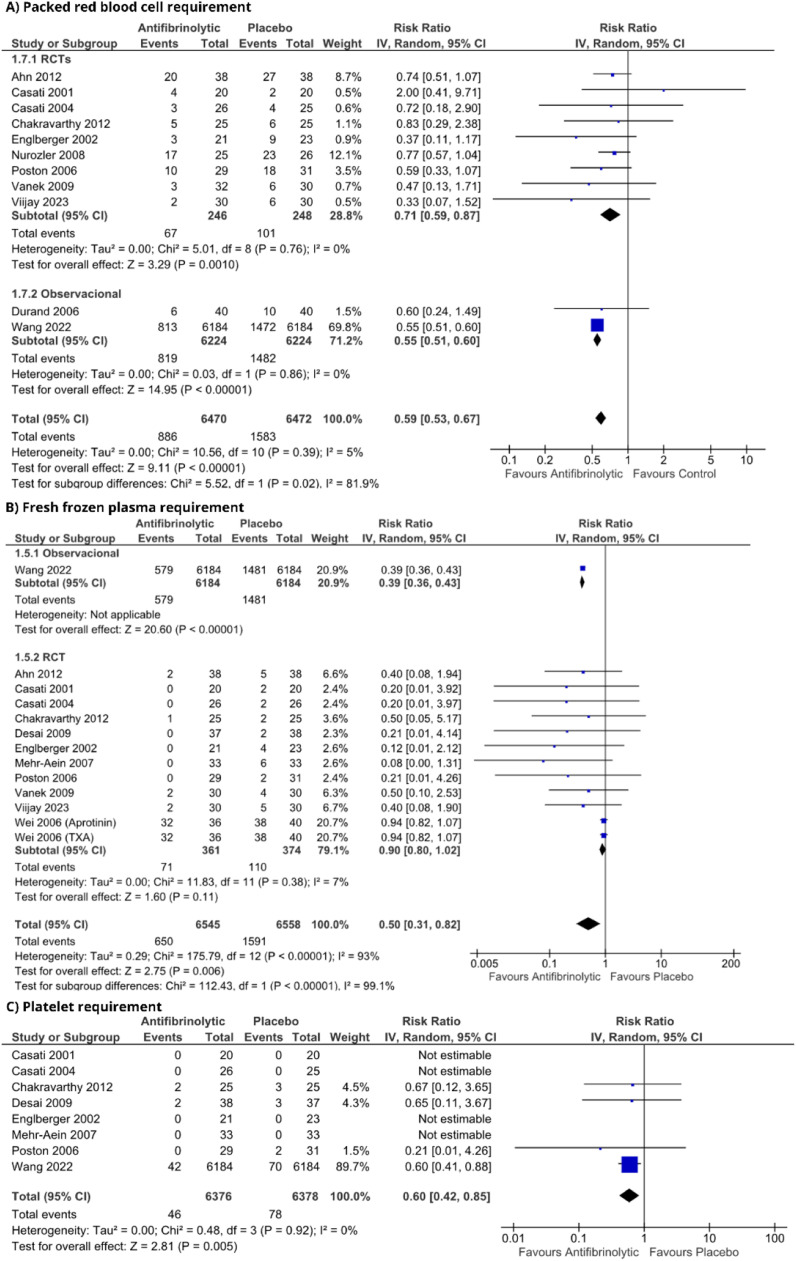
Blood transfusion requirement forest plots. (A) Packed red blood cell requirement; (B) Fresh frozen plasma requirement; (C) Platelet requirement. IV, Inverse Variance; RCT, Randomized Controlled Trial; CI, Confidence Interval.

**Figure 3:**
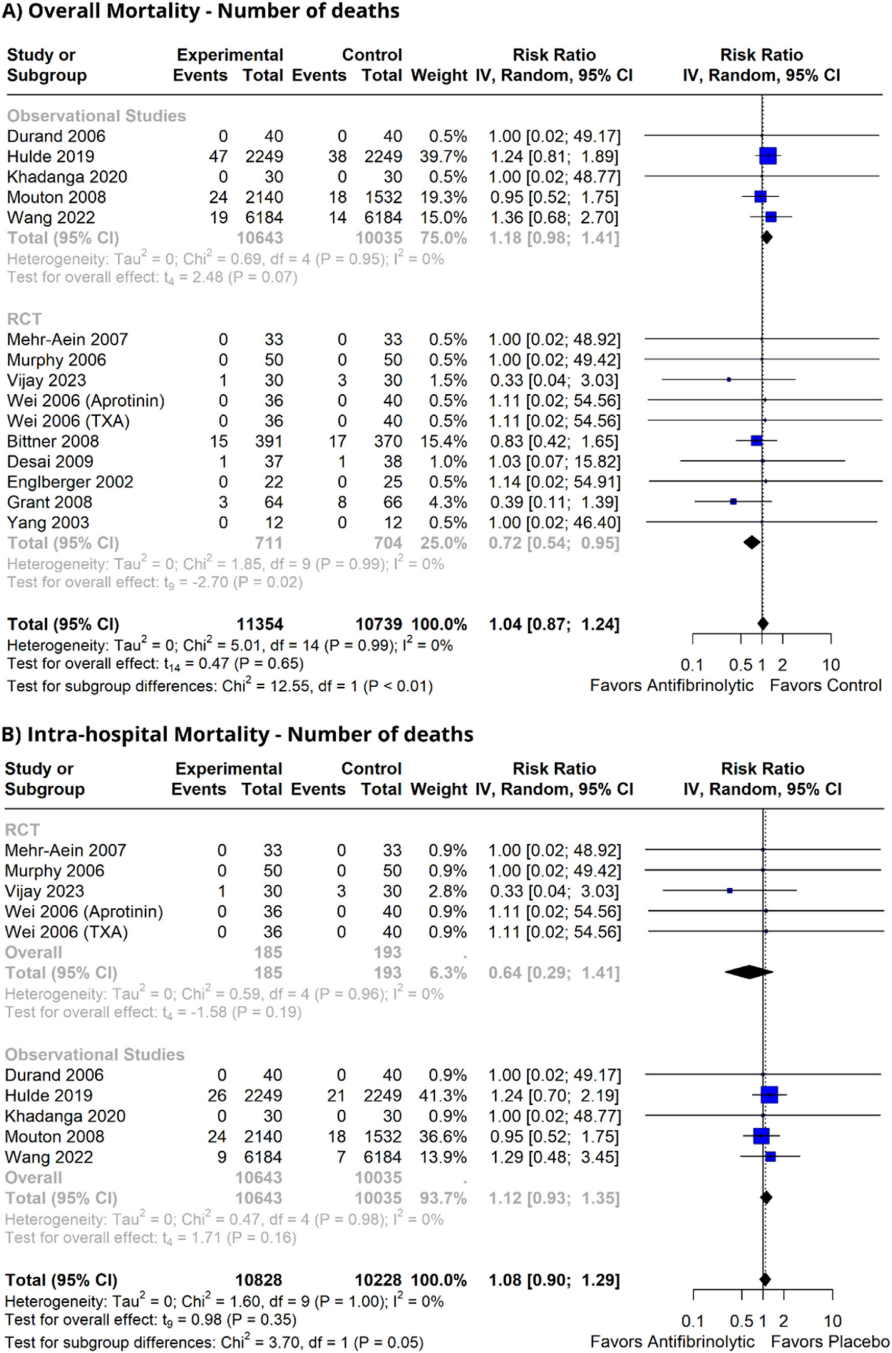
Overall mortality forest plots. Number of deaths. (A) Overall mortality with number of deaths. (B) Intra-hospital with number of deaths. IV, Inverse Variance; RCT, Randomized Controlled Trial; CI, Confidence Interval.

**Figure 4:**
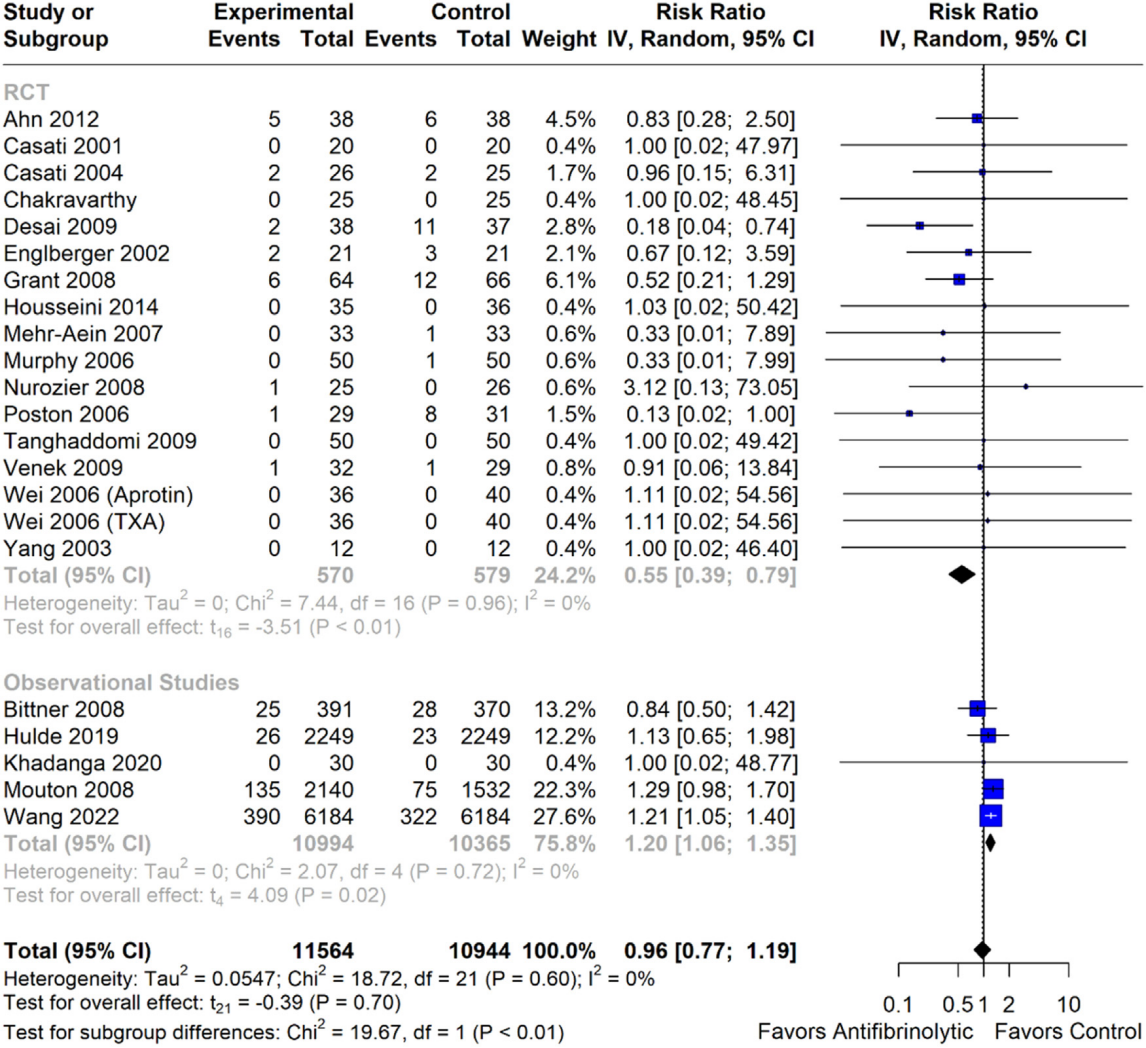
Thromboembolic events. Number of events. IV, Inverse Variance; RCT, Randomized Controlled Trial; CI, Confidence Interval.

Reduction of the intraoperative blood loss (MD = -17.47; [95% CI -77.82 to 42.88]; p > 0.05; I^2^ = 38%) or in time until the discharge of the intensive care unit (MD = 0.8; [95% CI -2.08 to 3.68]; p > 0.05; I^2^ = 89%) were not observed (Fig. S15). The number of patients who underwent packed red blood cell transfusion reduced significantly (RR = 0.59; [95% CI 0.53 to 0.67]; p < 0.05; I^2^ = 5%) similar to fresh frozen plasma (RR = 0.50; [95% CI 0.31 to 0.82); p < 0.05; I^2^ = 93%) and platelets (RR = 0.60; [95% CI 0.42 to 0.85]; p < 0.05; I^2^ = 0%) (Fig. 2). There was no change in either overall mortality (RR = 1.04; [95% CI 0.87 to 1.24]; p = 0.65; I^2^ = 0%) or in-hospital mortality (RR = 1.08; [95% CI 0.90 to 1.29]; p = 0.35; I^2^ = 0%] (Fig. 3). Regarding thromboembolic outcomes, a non-significant result was observed (RR = 0.96; [95% CI 0.77, 1.19]; p = 0.70; I^2^ = 0%] (Fig. 4).

### Subgroup analyses

The result of packed red blood cells was reduced in both randomized studies (RR = 0.74; [95% CI 0.60 to 0.91]; p < 0.05; I^2^ = 0%) and non-randomized studies (RR = 0.55; [95% CI 0.51 to 0.60]; p < 0.05; I^2^ = 0%). Intra-hospital mortality did not decrease in RCTs (RR = 0.64; [95% CI 0.29 to 1.41]; p = 0.54; I^2^ = 0%), unlike in non-RCTs (RR = 1.12; [95% CI 0.93 to 1.35]; p = 0.16; I^2^ = 0%). Overall mortality showed different results between RCTs (RR = 0.72; [95% CI 0.54 to 0.95]; p < 0.05; I^2^ = 0%) and observational studies (RR = 1.18; [95% CI 0.98; 1.41]; p = 0.07). Thromboembolic events showed a diverged result, with RCTs showing a reduction (RR = 0.55; [95% CI 0.39 to 0.79]; p < 0.05; I^2^ = 0%] and observational studies showing an increase (RR = 1.2; [95% CI 1.06; 1.35]; p = 0.02). Given the controversies surrounding the availability of aprotinin, a stratified analysis was performed across the antifibrinolytic agents: overall mortality was not reduced in RCTs for either TXA (RR = 0.58; [95% CI 0.20 to 1.65]; p = 0.19; I^2^ = 0%) or aprotinin (RR = 0.74; [95% CI 0.51 to 1.07]; p = 0.09; I^2^ = 0%) subgroups. In-hospital mortality was not reduced in RCTs for TXA (RR = 0.58; [95% CI 0.20 to 1.65]; p = 0.19; I^2^ = 0%]. Thromboembolic events were reduced in RCTs with aprotinin (RR = 0.44; [95% CI 0.23 to 0.86]; p = 0.02; I^2^ = 0%), while no changes were observed with TXA (RR = 0.78; [95% CI 0.60 to 1.01]; p = 0.05; I^2^ = 0%).

### Quality assessment

Rob-2 and ROBINS-I were used for quality assessment, as shown in Fig. S15. Three studies had a high risk of bias.^11^^,^^17^^,^^18^ Vijay et al.^18^ did not fully describe these methods. Durand et al.^11^ and Weingarten et al.^17^ reported a high bias due to participant selection, and only Weingarten et al.^17^ reported a high bias due to confounding factors. In the funnel plot analysis (Supplementary Appendix), studies occupied a symmetrical distribution according to weight and tended towards the pooled effect as their weight increased, except for the thromboembolic event outcome, which indicated a significant publication bias, confirmed by Egger’s test.

## Discussion

In this systematic review and meta-analysis of 27 studies and 23,149 patients, we compared antifibrinolytic use with placebo or no use during off-pump CABG. The leading findings with antifibrinolytics include: 1) A reduction in the need for blood component transfusions; 2) A lower risk of overall death in RCTs; 3) A lower incidence of thromboembolic events in RCTs; 4) No change in overall mortality across all studies; 5) No change in thromboembolic events in any study, and 6) A higher incidence of thromboembolic events in observational studies. These differences between RCTs and non-RCTs were confirmed via meta-regression (Supplementary Appendix).

CABG surgeries are associated with an increased risk of bleeding and the need for blood transfusions, with fibrinolysis playing an important role in this context.^19^ Antifibrinolytic agents such as TXA and aprotinin have a great capacity for hemostasis in cardiac surgery. However, when using these drugs, there is concern about an increased risk of thromboembolic events. The use of these agents in on-pump CABG is well established, which is not the case with off-pump CABG, for which it remains unclear whether the clinical outcomes are beneficial.^6^^,^^20^ A previous meta-analysis included only randomized studies in its methodology and lacked statistical power to determine the results, especially regarding postoperative thromboembolic complications, combining thromboembolic events and mortality into a single composite outcome.^7^ This study aimed to carry out a more comprehensive analysis that allows for the comparison of observational studies, including a larger patient number, with RCTs, while also investigating the effects of the antifibrinolytic class beyond tranexamic acid. The inclusion of observational studies in this meta-analysis was based on methodological and clinical considerations. First, the RCTs available on the use of antifibrinolytic agents in off-pump CABG involved relatively small sample sizes, limiting statistical power, particularly for rare outcomes such as thromboembolic events. Moreover, their inclusion enables data capture from more heterogeneous populations and diverse clinical settings, thus reflecting better real-world practice than the more restrictive inclusion criteria of RCTs. This approach also made it possible to assess consistency across different study designs, identify potential sources of heterogeneity, and address evidence gaps in outcomes or subgroups that are underrepresented in RCTs. Wang et al.^20^ present us with the analysis of 18,380 patients in a retrospective cohort from 2009 to 2019, highlighting this importance.

The results of this meta-analysis are consistent with those of previous meta-analyses regarding the hemostatic potential of antifibrinolytics and their ability to reduce blood product transfusions.^7^^,^^21^ However, the relationship between their use and thromboembolic outcomes or mortality was a new milestone in this study. The subgroup analysis of RCTs demonstrated a significant reduction in overall mortality, as well as a significant reduction in thromboembolic events. It is important to highlight that overall mortality represents an outcome with a lower level of bias than thromboembolic events in this study, since it is less heterogeneous. Mortality was assessed in a substantial proportion of the included studies with the assurance that it was evaluated during patient follow-up; however, the assessment and reporting of thromboembolic events remained unclear in the descriptions of the included studies.

It is important to emphasize that the small sample sizes in these studies and the rarity of the outcome required the use of a composite outcome in thromboembolic events. This composite grouped endpoints such as stroke, renal ischemia, pulmonary embolism, myocardial ischemia, deep vein thrombosis, and major neurological dysfunction across the analyzed studies. When comparing the outcomes of thromboembolic events, substantial heterogeneity was observed in the assessments. Some studies evaluated complications only within the first 24 hours, such as Casati et al.^22^ and Casati et al.,^23^ whereas others, such as Desai et al.,^12^ assessed complications up to 72 hours postoperatively, illustrating differences in follow-up duration. Moreover, different composite outcomes involving thromboembolic events were present within individual studies, as in Grant et al.^24^ Additionally, significant publication bias was identified, further complicating the analysis of the results herein reported for this outcome, which should be interpreted with caution. There was also heterogeneity and lack of information regarding the diagnostic criteria for each complication, as most dealt with clinical subjectivity, such as pulmonary embolism and renal dysfunction in Mehr-Aein et al.,^25^ or cerebral complications, as reported by Wei et al.^26^ Consequently, there was heterogeneity in the diagnostic criteria, thromboembolic outcomes assessed, or follow-up period. For a more thorough analysis, in an attempt to decrease residual heterogeneity, the thromboembolic outcomes were stratified according to the type of composite outcome employed in each primary investigation, revealing results consistent with those observed in the non-stratified analyses, as shown in the Supplementary Materials.

Consequently, we observed discrepancies wherein observational studies reported distinct findings regarding overall mortality and divergent results for thromboembolic events. Several hypotheses have been proposed to explain these discrepancies. First, the study by Wang et al.,^20^ in the sensitivity analysis presented in the Supplementary Material, showed a strong influence on increasing the risk ratio for thromboembolic events. In addition, the lack of methodological robustness and homogeneity in the assessment of thromboembolic outcomes across the included studies introduced potential biases that could have led to misleading results, thereby warranting cautious finding interpretations. Additionally, the RCTs included were prospective in design but generally featured relatively short follow-up periods compared to retrospective observational studies, which are better suited for capturing long-term morbidity outcomes. Lastly, given that thromboembolic events have relatively low-incidence outcomes and that RCTs had relatively limited sample sizes, the substantially larger patient populations in observational studies increase the likelihood of detecting these events. This disparity in sample size may partly explain the higher incidence of thromboembolic events reported by observational cohorts. Taken together, these factors underscore the need for careful consideration when comparing results across study designs and highlight the importance of standardized follow-ups in future research.

Observational studies evaluating antifibrinolytic use are susceptible to confounding by indication, wherein treatment assignment is influenced by a patient's baseline characteristics and prognosis. Institutional protocols or clinician judgment may lead to sicker patients at a higher risk of bleeding being more likely to receive an antifibrinolytic, or conversely, patients with a high risk of thrombosis or renal injury being more likely to avoid them. This can introduce a significant bias, potentially skewing the results. To mitigate this, the observational estimates included in this review were adjusted using various statistical methods. Several of the larger retrospective studies explicitly used Propensity Score Matching (PSM) or propensity-adjusted multivariate logistic regression to balance dozens of baseline covariates between the treatment and control groups, thereby creating more comparable cohorts for analysis. For example, Wang et al.^20^ used PSM to balance 32 patient and treatment factors, including comorbidities, preoperative medications, and surgical variables. Other studies used direct matching on a smaller set of variables such as age and gender or presented unadjusted estimates while acknowledging the lack of control for confounding as a major limitation. It is important to emphasize that, despite the limitations of unadjusted characteristics, patients undergoing this type of procedure have similar medical profiles, with cardiovascular comorbidities and advanced age.

Despite the presence of well-designed randomized studies with a low risk of bias, analyzing the off-pump CABG management landscape presents inherent challenges when comparing different articles. Significant heterogeneity was evident in intraoperative blood loss comparisons, which may be attributed to calculation methods and biases introduced by heterogeneous team preferences, such as varying degrees of accidental fluid mixing during aspiration or blood absorption by clothes from surgical drapes and gauzes. Therefore, the outcomes of blood product transfusions were used. This provides greater precision and objectivity in assessing the level of blood loss than measuring blood loss alone. Moreover, it serves as a clinically significant marker because excessive bleeding is often accompanied by the need for transfusion. Additionally, dosing regimens varied across studies despite commonly involving an initial bolus followed by continuous infusion, adding another layer of uncertainty to the analysis.

Furthermore, there were differences in how the studies defined transfusion triggers for blood products, with each medical service applying its own criteria (Supplementary Table S1). For example, Vanek et al.^27^ initiated red blood cell transfusion when hemoglobin dropped below 8.5 g.dL^-1^ and/or the hematocrit level, while transfusion of fresh frozen plasma was initiated to correct suspected coagulation factor deficiencies when chest drain bleeding exceeded > 150 mL.h^-1^ or > 100 mL.h^-1^ for two consecutive hours. Casati et al.,^23^ on the other hand, used the presence of hypovolemia signs or symptoms (hypotension or tachycardia) or diffuse bleeding as criteria for transfusion.

The use of systemic aprotinin is limited by critical safety issues, primarily increased mortality risk as demonstrated by the BART clinical trial, which compared it to safer lysine analogues.^28^ These adverse findings led to a global market suspension of the drug in 2007. Within this context, a stratified analysis of the results between aprotinin and lysine analogs was performed. Regarding overall mortality, although individual RCTs investigating TXA and aprotinin did not show a reduction, decreased overall risk was observed when the subgroups were pooled, as shown in Supplementary Figure S6. This finding raises hypotheses to be considered, such as the limited sample sizes and the heterogeneity of institutional protocols. Confirming previous evidence, for in-hospital mortality, there was increased risk of death in the aprotinin subgroup, although the number of available studies limits data reliability; and it should be noted that only one RCT evaluating aprotinin for in-hospital mortality was available, whereas the other two trials were observational. For thrombotic events, RCTs employing aprotinin demonstrated a risk reduction, whereas RCTs which used TXA showed a borderline result that warrants watchful interpretation. The analysis suggested a trend toward reduced risk (RR = 0.78; 95% CI 0.60–1.01; p = 0.055), although the confidence interval indicated non-significance. The absence of heterogeneity (I^2^ = 0%) supports consistency across studies, although there were few occurrences of the outcome and the borderline p-value raises concerns regarding the robustness of the finding. Conversely, the analysis of observational studies employing TXA found increased risk of thrombotic events. As such, there is significant heterogeneity in the current literature and caution must be exerted when interpreting the results. All meta-analyses involving the stratification of antifibrinolytics by outcome can be accessed in the Supplementary Material.

The present study had several limitations. However, these were not sufficient to discredit the findings of our study, as all analyzed outcomes, except ICU stay duration, fresh frozen plasma requirement, and intraoperative blood loss, showed low calculated heterogeneity. Of the 27 included studies, 20 were randomized^9^^,^^12^^,^^18^^,^^22^, ^23^, ^24^, ^25^, ^26^, ^27^^,^^29^, ^30^, ^31^, ^32^, ^33^, ^34^, ^35^, ^36^, ^37^, ^38^ and seven were observational.^11^^,^^17^^,^^20^^,^^39^, ^40^, ^41^, ^42^ To minimize the bias introduced by including nonrandomized studies, we conducted several sub-analyses of their results, which provided a better understanding of their effect on the overall sample. This approach allowed the comparison of a large patient population derived from these studies with a smaller population of randomized studies, thereby enabling hypothesis generation to explain the differences and discrepancies between the methodologies. This finding highlights the need for further analysis in yet-to-be published studies.

Finally, an overall optimistic safety profile emerges for the use of antifibrinolytics in off-pump CABG. Reductions in mortality, thromboembolic outcomes, and the need for blood product transfusion suggest tangible benefits for both patients and the health system. Nevertheless, the evidence base would be strengthened by additional, adequately powered, randomized trials with a more standardized assessment of thromboembolic events and mortality. Notably, the perioperative efficacy of these agents in lowering transfusion requirements is well established.^4^^,^^5^

## Conclusion

This updated and comprehensive meta-analysis encompassing 23,149 patients presents results consistent with the literature regarding reduction in the need for blood product transfusion, introducing a reduction in overall mortality and thromboembolic events in RCT subgroups as novel findings. Clinical recommendations should focus primarily on lysine analogues, tranexamic acid and ε-aminocaproic acid, not aprotinin. Discrepancies were found when RCT and non-RCT subgroups were compared for thromboembolic events, which may be explained by the previously discussed hypotheses involving heterogeneity and composite outcome limitations. Whereas observational data do not support such reductions in some cases and may suggest harm, hence, clinical decisions should privilege RCT-derived estimates. Further large, contemporary, standardized randomized studies with harmonized thromboembolic definitions are necessary to clarify the outcomes of mortality and thromboembolic events, considering the efficacy and safety benefits observed to date.

## Conflicts of interest

The authors declare no conflicts of interest.

## Appendix Supplementary materials

Image, application 1

## Footnotes

Supplementary material associated with this article can be found in the online version at doi:10.1016/j.bjane.2026.844731.
