# Supplementary material for: Effectiveness and safety of antifibrinolytic agents in off-pump coronary artery bypass grafting: a systematic review and meta-analysis
Source: Braz J Anesthesiol. 2026 Jan 24;76(2):844731. doi: 10.1016/j.bjane.2026.844731 (PMC12992088; doi:10.1016/j.bjane.2026.844731)
Supplement: Supplementary file 1 [file mmc1.pdf]

S1) Transfusion Criteria

Study	Red blood cells	Fresh frozen plasma	Platelets
Ahn, 2012	Not specified; transfusion given per clinical assessment.	Not specified.	Not specified.
Bert, 2008	Not specified.	Not specified.	Not specified.
Bittner, 2008	Indications included hematocrit < 25% or clinical instability (e.g., hemodynamic instability/tachycardia); elderly age considered.	Not specified.	Not specified.
Casati, 2001	PRBC if hemoglobin < 8 g/dL or hematocrit < 24% with signs/symptoms of hypovolemia (hypotension and/or tachicardia).	Infuse if PT after protamine administration ≥ 1.5 times than the baseline, with diffuse bleeding.	Transfuse if platelet count ≤ 50,000/mm³ with diffuse bleeding.
Casati, 2004	PRBC if hemoglobin < 8 g/dL or hematocrit < 24%.	Infuse if PT after protamine administration ≥ 1.5 times than the baseline, with diffuse bleeding.	Transfuse if platelet count ≤ 50,000/mm³.
Chakravarthy, 2012	Transfusion threshold hemoglobin = 9 g/dL.	Not specified.	Not specified.
Desai, 2009	Not specified.	Not specified.	Not specified.
Durand, 2006	Not specified.	Not specified.	Not specified.
Englberger, 2002	Not specified.	Not specified.	Not specified.
Grant, 2008	Not specified.	Not specified.	Not specified.
Hosseini, 2014	Transfuse if HCT < 24% and Hb < 8 g/dL.	FFP if PT ≥ 1.5 times than the basic rate with disseminated bleeding.	Platelets if disseminated bleeding and platelet count < 50,000/mm³.
Hulde, 2019	Not specified.	Not specified.	Not specified.
Khandanga et al. (2020)	Not specified.	Not specified.	Not specified.
Kim, 2004	Not specified (transfusions reported but no explicit thresholds).	Not specified.	Not specified.
Mehr-Aein, 2007	Transfusion if HCT < 20% and/or Hb < 7 g/dL during the operation; and HCT < 28% and/or Hb < 9 g/dL postoperatively.	FFP if PT > 1.5 times than the normal range and bleeding > 200 mL/h.	Platelets if count < 75×10⁹/L and bleeding > 200 mL for longer than 2 hr.
Mouton, 2008	Not specified.	Not specified.	Not specified.
Murphy, 2006	Not specified.	Not specified.	Not specified.
Nurozler et al., 2008	PRBC if hematocrit < 25%.	FFP if chest tube drainage > 250 mL/h after the 1st hour, despite normalized ACT.	Platelets perioperatively if platelet count < 80,000/mL or pathologic bleeding time > 10 min; also considered with high drainage per ICU criterion.
Poston et al., 2006	Not specified.	Not specified.	Not specified.
Taghaddomi, 2009	PRBC if hemoglobin < 9 g/dL and/or hematocrit < 27%.	FFP if chest tube bleeding > 150 mL/h or > 100 mL/h for two consecutive hours.	Not specified.
Vanek et al., 2005	PRBC if hemoglobin < 8.5 g/dL and/or hematocrit < 26%.	FFP if chest drain bleeding > 150 mL/h or > 100 mL/h for two consecutive hours.	Not specified.
Vijay et al., 2023	Not specified.	Not specified.	Not specified.
Wang et al., 2022	Not specified.	Not specified.	Not specified.
Wei et al., 2006	Whole blood when hemoglobin < 85 g/L.	FFP to correct suspected coagulation factor deficiency or maintain suitable circulating volume.	Not specified.
Wei et al., 2006	Whole blood when hemoglobin < 85 g/L.	FFP to correct suspected coagulation factor deficiency or maintain suitable circulating volume.	Not specified.
Weingarten et al., 2021	Not specified.	Not specified.	Not specified.
Yan et al., 2003	Not specified.	Not specified.	Not specified.

Table S1. Transfusion criteria of included studies. RBC, red blood cell; PRBC packed red blood cell; FFP, fresh frozen plasma; HCT, hematocrit; Hb, hemoglobin; PT, prothrombin time; ACT, activated clotting time; ICU, intensive care unit.

S2) Overall Mortality

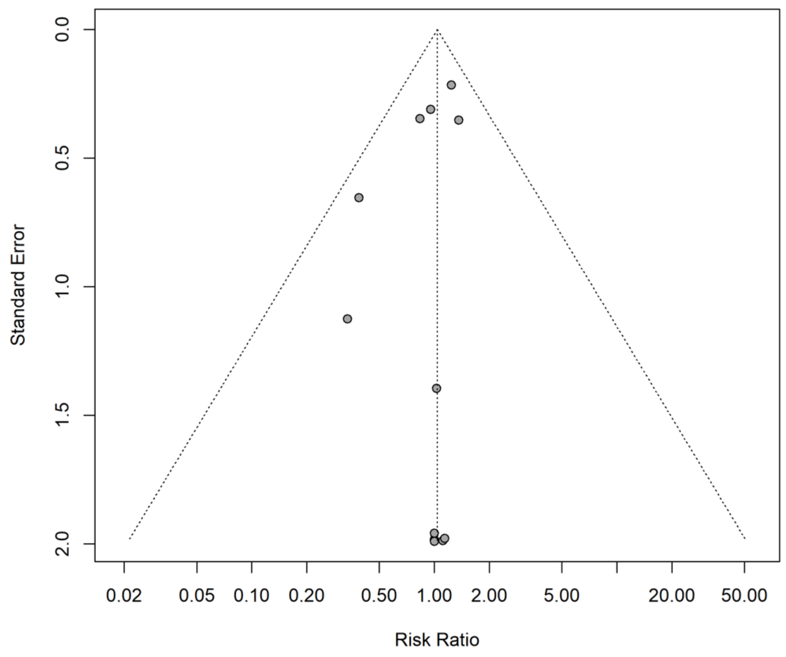

Egger's test for General Mortality: Intercept [CI] = -0.26 [-0.7; 0.17]; p-value = 0.258

S3)Thromboembolic Events

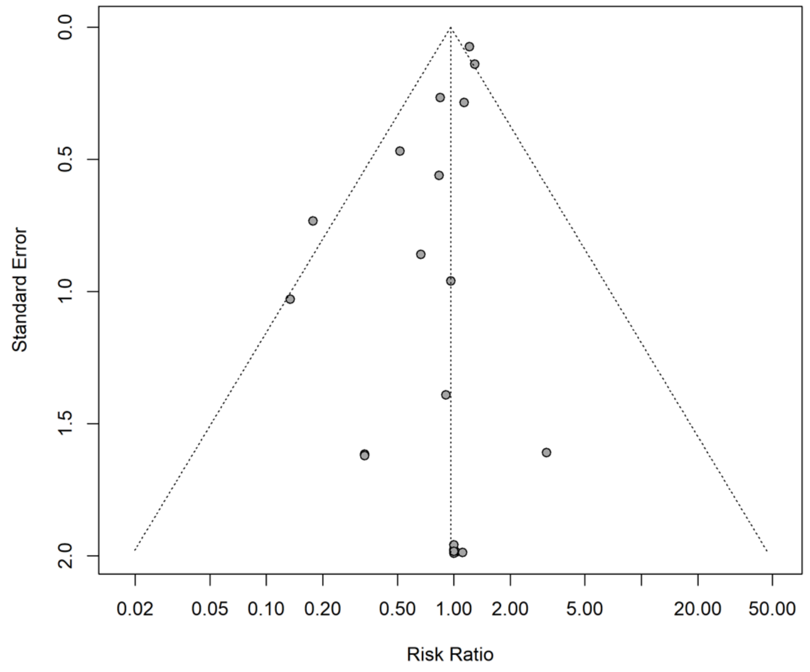

Egger's test for Thrombotic Events: Intercept [CI] = -0.57 [-0.99; -0.16]; p-value = 0.013*

S4) Intra-hospital mortality

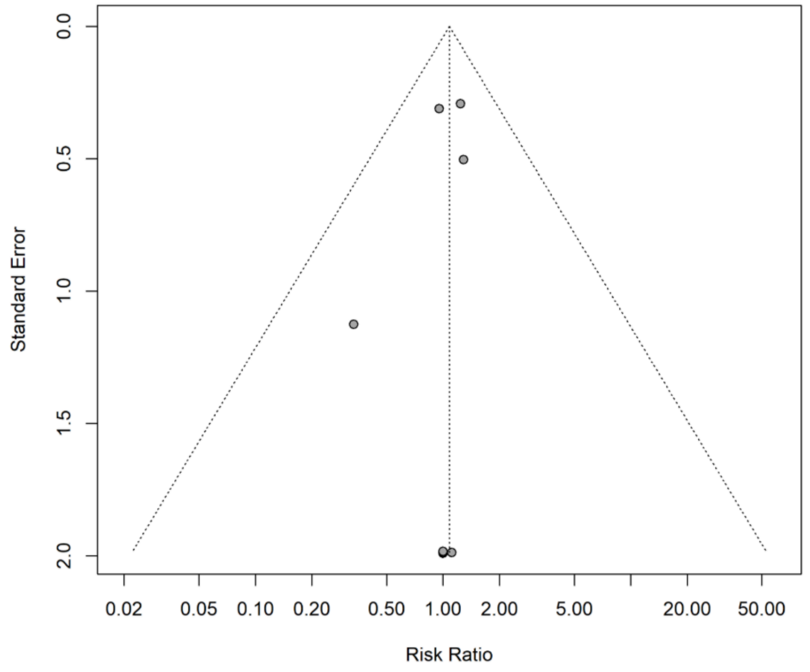

Egger's test for Intrahospital Mortality: Intercept [CI] = -0.17 [-0.57; 0.23]; p-value = 0.423

S5)Sensitivity Analysis - Removed Wang et al. 2022

Sensitivity Analysis - removed: Wang et al. 2022

A) General Mortality

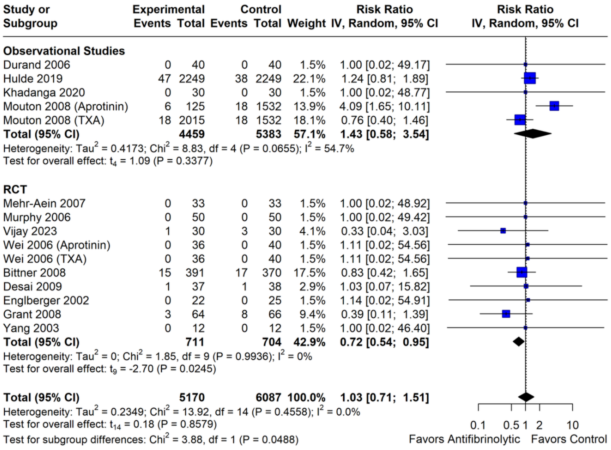

B) In-Hospital Mortality

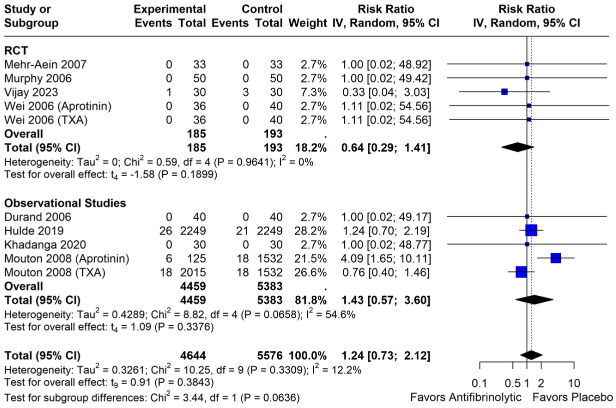

C) Thrombotic Events

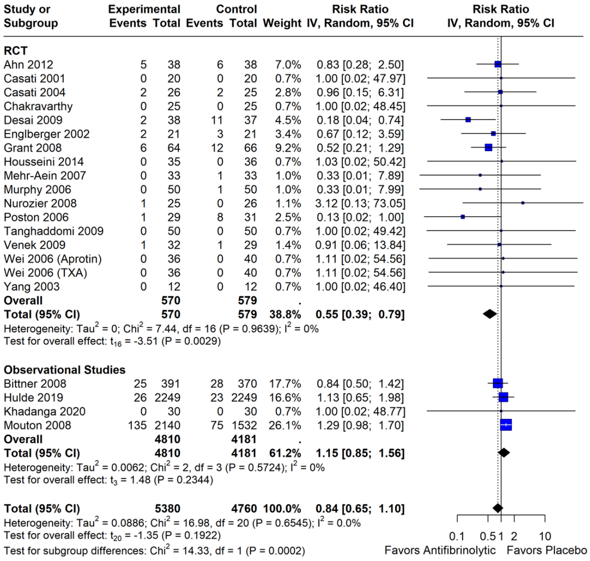

S6) TXA versus Aprotinin Comparison - Overall Mortality

Overall Mortality - Forest Plot - Additional Analyses

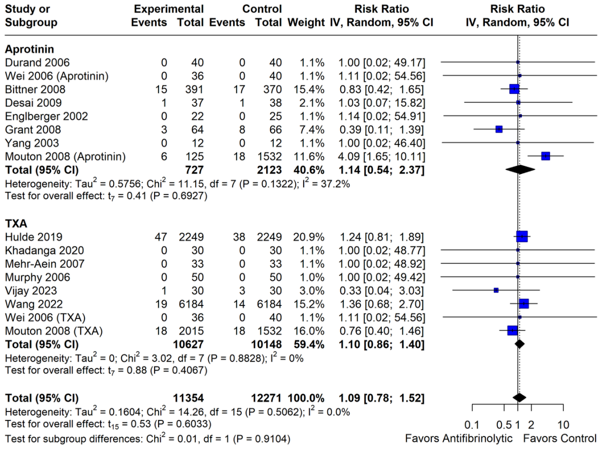

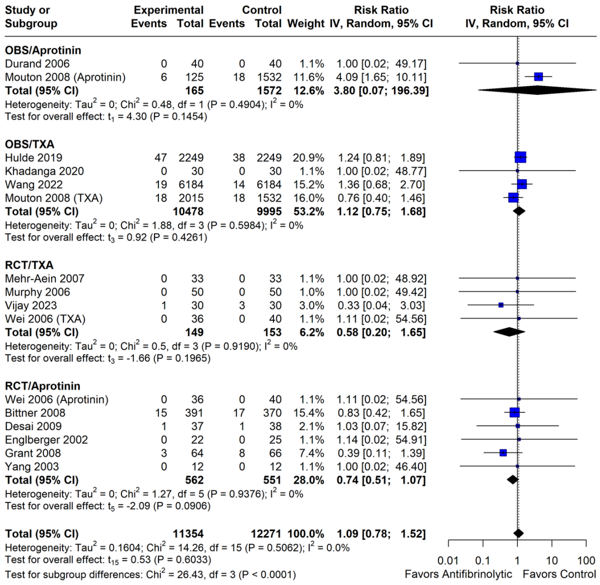

S7) TXA versus Aprotinin Comparison - In-Hospital Mortality

In-Hospital Mortality - Forest Plot - Additional Analyses

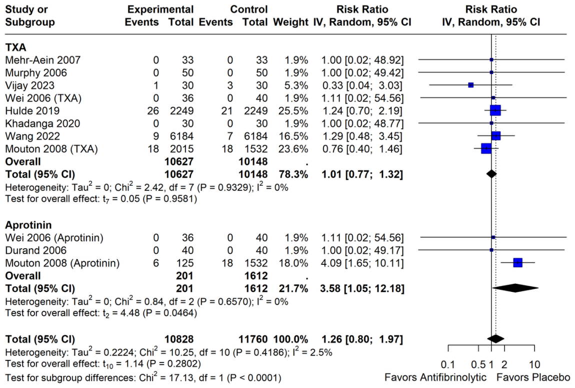

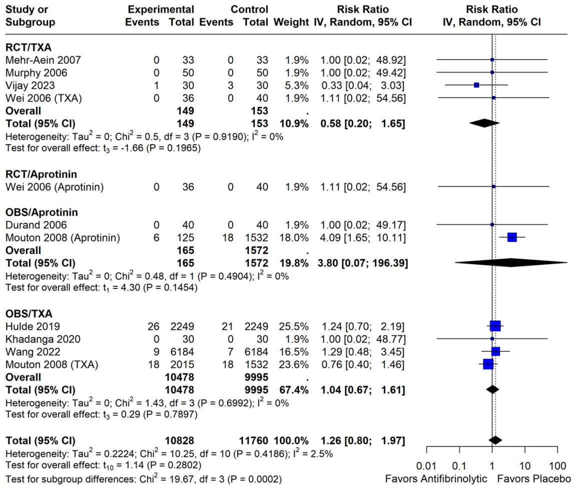

S8) TXA versus Aprotinin Comparison - Thrombotic Events

Additional Analyses: Thrombotic Events

A) TXA versus Aprotinin

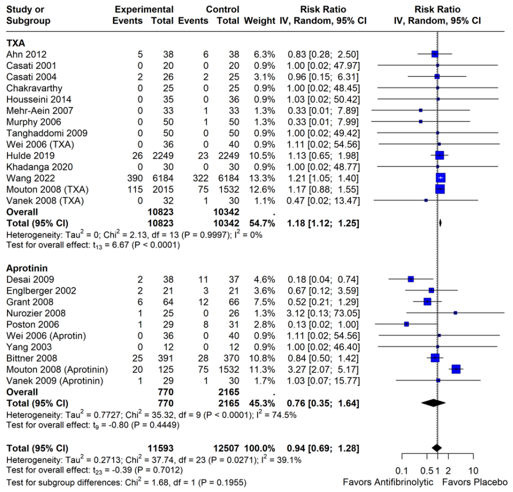

B) Type of composite endpoints

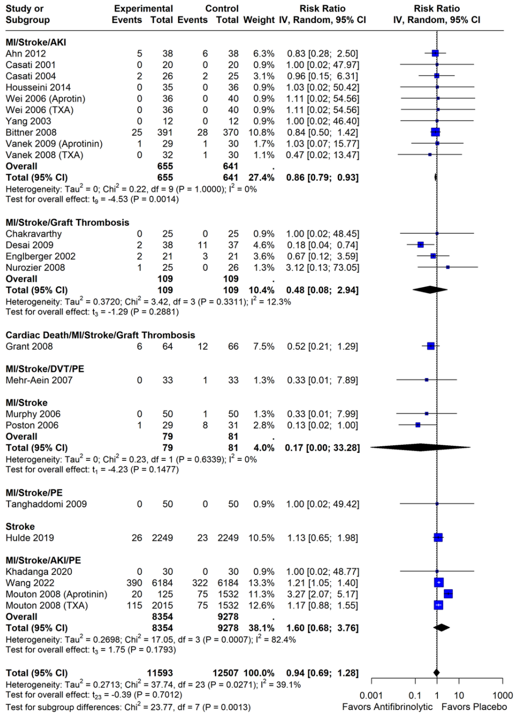

C) Type of Antifibrinolytic and Design

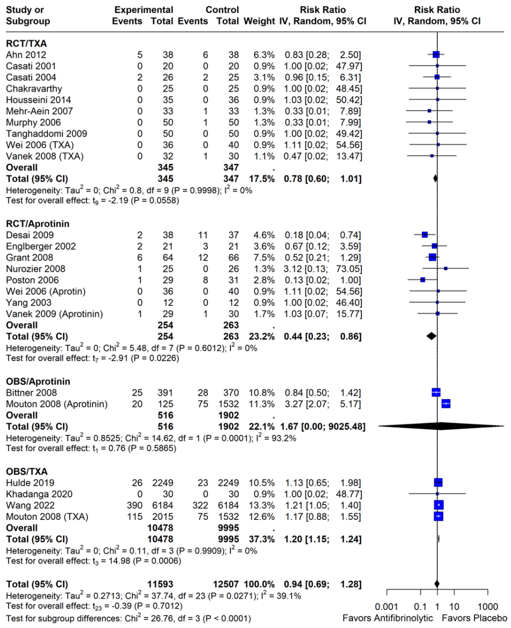

Legend:

MI - Myocardial Infarction
AKI - Acute Kidney Injury
PE - Pulmonary Embolism
DVT - Deep Vein Thrombosis
TXA - Tranexamic Acid
RCT - Randomized Controlled Trials
OBS - Observational Studies

S9) Meta-regression: Quantitative effect of study design over the primary outcomes of the review

General Mortality

Variable	Estimate [CI]	p-value
RCTs	-0.33 [-0.59; -0.07]	0.016
Observational Studies	0.16 [0.01; 0.31]	0.036

Thrombotic Events

Variable	Estimate [CI]	p-value
RCTs	-0.59 [-0.94; -0.23]	0.002
Observational Studies	0.18 [0.09; 0.26]	< 0.001

Intrahospital Mortality

Variable	Estimate [CI]	p-value
RCTs	-0.45 [-1.07; 0.17]	0.146
Observational Studies	0.11 [-0.04; 0.27]	0.133

Thus, study design was significantly associated with differences in the effect of general mortality and thrombotic events, and the association occurred in the same direction.

RCTs were associated with smaller (more negative) effect measures (RR), whereas observational studies reported less negative effects. This means that for the two outcomes, RCTs tended to favor antifibrinolytics more than Observational Studies).

S10) TXA - dose, route and timing

Study	TXA Dose (total mg or mg/kg)	Route	Timing
Ahn, 2012	1 g bolus + 200 mg/h infusion	IV bolus + continuous infusion	Before incision, continued perioperatively
Bert, 2008	Not applicable (Aprotinin)	IV bolus + infusion	During surgery until skin closure
Bittner, 2008	Not applicable (Aprotinin)	IV bolus (1–2 million KIU)	At sternotomy, intraoperative
Casati, 2001	1 g bolus + 400 mg/h infusion	IV bolus + continuous infusion	Before incision, intraoperative
Casati, 2004	1 g bolus + 400 mg/h infusion (+500 mg in CPB)	IV bolus + continuous infusion	Before incision, intraoperative
Chakravarthy, 2012	10 mg/kg over 30 min + 1 mg/kg/h for 12h	IV bolus + continuous infusion	Perioperative, intra + 12h postop
Desai, 2009	Not applicable (Aprotinin)	IV bolus + continuous infusion	During OPCAB surgery
Durand, 2006	Not applicable (Aprotinin)	IV bolus + continuous infusion	During OPCAB surgery
Englberger, 2002	Not applicable (Aprotinin)	IV bolus + continuous infusion	During OPCAB surgery
Grant, 2008	Not applicable (Aprotinin)	IV bolus + continuous infusion	During OPCAB surgery
Hosseini, 2014	1 g topical in 100 mL saline applied to pericardium	Topical application	Applied at end of surgery (topical)
Hulde, 2019	1 g IV single bolus	IV bolus	After heparinization, intraoperative
Khadanga, 2020	10 mg/kg IV bolus at induction (single dose)	IV bolus	At induction of anesthesia
Kim, 2004	10 mg/kg IV bolus before incision + 1 mg/kg/h infusion	IV bolus + continuous infusion	Bolus before incision, infusion during surgery
Mehr-Aein, 2007	15 mg/kg before heparin + 15 mg/kg after protamine	IV bolus	At beginning and end of surgery
Mouton, 2008	Not applicable (Aprotinin)	IV bolus + infusion (Aprotinin)	During surgery (observational)
Murphy, 2006	2 g IV bolus before sternotomy	IV bolus	Before sternotomy
Nurozler, 2008	Not applicable (Aprotinin, low-dose)	IV bolus + infusion (Aprotinin)	During surgery until end
Poston, 2006	Not applicable (Aprotinin, 2 million KIU + 0.5 million KIU/h)	IV bolus + continuous infusion	Before sternotomy, maintained until end
Taghaddomi, 2009	1 g IV bolus before incision + 200 mg/h infusion	IV bolus + continuous infusion	Before incision + intraoperative infusion
Vanek, 2005	1 g IV bolus before incision + 200 mg/h infusion (TXA group)	IV bolus + continuous infusion	Before incision + intraoperative infusion
Vijay, 2023	15 mg/kg IV before surgery + 15 mg/kg at end of surgery	IV bolus (two doses)	Before surgery + at end of surgery
Wang, 2022	15 mg/kg IV bolus at induction + 10 mg/kg/h infusion	IV bolus + continuous infusion	Bolus at induction, infusion maintained intraoperatively
Wei, 2006	0.75 g IV loading dose + 0.25 g/h infusion (total ~1.5 g)	IV bolus + continuous infusion	Bolus at beginning of surgery, infusion throughout
Wei, 2006	0.75 g IV loading dose + 0.25 g/h infusion (total ~1.5 g)	IV bolus + continuous infusion	Bolus at beginning of surgery, infusion throughout
Weingarten, 2021	Mean 2.1 g IV (SD 1.5 g), variable bolus/infusion regimens	IV (bolus ± infusion, chart review)	Perioperative administration (before/during OPCAB)
Yang, 2003	Not applicable (Aprotinin study)	IV bolus + infusion (Aprotinin)	During OPCAB surgery

Table S10. Dose, route and timing of TXA in selected studies. TXA, Tranexamic Acid; OPCAB, Off-Pump Coronary Artery Bypass; CPB, Cardiopulmonary Bypass; SD, Standart Deviation.

S11) Aprotinin - dose, route and timing

Study	Aprotinin Dose (KIU)	Route	Timing
Ahn, 2012	Not applicable (no aprotinin arm)	-	-
Bert, 2008	Not reported	Not reported	Perioperative/intraoperative
Bittner, 2008	Not reported	Not reported	Perioperative/intraoperative
Casati, 2001	Not applicable (no aprotinin arm)	-	-
Casati, 2004	Not applicable (no aprotinin arm)	-	-
Chakravarthy, 2012	Not applicable (no aprotinin arm)	-	-
Desai, 2009	2M KIU IV bolus + 0.5M KIU/h infusion	IV bolus + continuous infusion	Bolus at incision; intraoperative infusion until end of surgery
Durand, 2006	Not reported	Not reported	Perioperative/intraoperative
Englberger, 2002	Not reported	Not reported	Perioperative/intraoperative
Grant, 2008	Not reported	Not reported	Perioperative/intraoperative
Hosseini, 2014	Not applicable (no aprotinin arm)	-	-
Hulde, 2019	Not applicable (no aprotinin arm)	-	-
Khadanga, 2020	Not applicable (no aprotinin arm)	-	-
Kim, 2004	1M KIU IV bolus before incision + 0.25M KIU/h intraoperative infusion	IV bolus + continuous infusion	Bolus at incision; infusion throughout OPCAB
Mehr-Aein, 2007	Not applicable (no aprotinin arm)	-	-
Mouton, 2008	Not reported	Not reported	Perioperative/intraoperative
Murphy, 2006	Not applicable (no aprotinin arm)	-	-
Nurozler, 2008	1M KIU IV bolus before incision + 0.25M KIU/h intraoperative infusion	IV bolus + continuous infusion	Bolus at incision; infusion throughout OPCAB
Poston, 2006	2M KIU IV bolus + 0.5M KIU/h infusion (Full-dose)	IV bolus + continuous infusion	Bolus at sternotomy; intraoperative infusion until end of surgery
Taghaddomi, 2009	Not applicable (no aprotinin arm)	-	-
Vanek, 2005	1M KIU IV bolus before incision + 0.25M KIU/h intraoperative infusion	IV bolus + continuous infusion	Bolus at incision; infusion throughout OPCAB
Vijay, 2023	Not applicable (no aprotinin arm)	-	-
Wang, 2022	Not applicable (no aprotinin arm)	-	-
Wei, 2006	1M KIU IV bolus before incision + 0.25M KIU/h intraoperative infusion	IV bolus + continuous infusion	Bolus at incision; infusion throughout OPCAB
Wei, 2006	1M KIU IV bolus before incision + 0.25M KIU/h intraoperative infusion	IV bolus + continuous infusion	Bolus at incision; infusion throughout OPCAB
Weingarten, 2021	Not applicable (no aprotinin arm)	-	-
Yang, 2003	Not reported	Not reported	Perioperative/intraoperative

Table S11. Dose, route and timing of aprotinin in selected studies. OPCAB, Off-Pump Coronary Artery Bypass.

S12) Anticoagulation Protocol

Study	Anticoagulation protocol	Protocol details
Ahn, 2012	Reported	Heparin 300 U/kg IV, maintain ACT > 300 s, reversal with protamine (1 mg per 100 U heparin)
Bert, 2008	Reported	Heparin 300 U/kg IV, maintain ACT > 300 s, reversal with protamine (1 mg per 100 U heparin)
Bittner, 2008	Reported	Heparinization with UFH, ACT target > 300 s, reversed with protamine; perioperative aspirin/clopidogrel management described.
Casati, 2001	Reported	Heparin 300 U/kg IV, maintain ACT > 300 s, reversal with protamine (1 mg per 100 U heparin)
Casati, 2004	Reported	UFH 150–200 U/kg IV, maintain ACT ≥ 300 s during OPCAB, reversed with protamine; perioperative aspirin management reported.
Chakravarthy, 2012	Reported	Heparin 300 U/kg IV, maintain ACT > 300 s, reversal with protamine (1 mg per 100 U heparin)
Desai, 2009	Reported	Heparin 300 U/kg IV, maintain ACT > 300 s, reversal with protamine (1 mg per 100 U heparin)
Durand, 2006	Reported	Heparin 300 U/kg IV, maintain ACT > 300 s, reversal with protamine (1 mg per 100 U heparin)
Englberger, 2002	Reported	UFH 300 U/kg IV bolus, maintain ACT ≥ 300 s intraoperatively, reversed with protamine; all patients on perioperative aspirin.
Grant, 2008	Reported	Heparin 300 U/kg IV, maintain ACT > 300 s, reversal with protamine (1 mg per 100 U heparin)
Hosseini, 2014	Reported	Heparin 300 U/kg IV, maintain ACT > 300 s, reversal with protamine (1 mg per 100 U heparin)
Hulde, 2019	Reported	Heparin 300 U/kg IV, maintain ACT > 300 s, reversal with protamine (1 mg per 100 U heparin)
Khadanga, 2020	Reported	Heparin 300 U/kg IV, maintain ACT > 300 s, reversal with protamine (1 mg per 100 U heparin)
Kim, 2004	Reported	Heparin 300 U/kg IV, maintain ACT > 300 s, reversal with protamine (1 mg per 100 U heparin)
Mehr-Aein, 2007	Reported	Heparin 300 U/kg IV, maintain ACT > 300 s, reversal with protamine (1 mg per 100 U heparin)
Mouton, 2008	Reported	Heparin 300 U/kg IV, maintain ACT > 300 s, reversal with protamine (1 mg per 100 U heparin)
Murphy, 2006	Reported	Heparin 300 U/kg IV, maintain ACT > 300 s, reversal with protamine (1 mg per 100 U heparin)
Nurozler, 2008	Reported	Heparin 300 U/kg IV, maintain ACT > 300 s, reversal with protamine (1 mg per 100 U heparin)
Poston, 2006	Reported	Heparin 300 U/kg IV, maintain ACT > 300 s, reversal with protamine (1 mg per 100 U heparin)
Taghaddomi, 2009	Reported	Heparin 300 U/kg IV, maintain ACT > 300 s, reversal with protamine (1 mg per 100 U heparin)
Vanek, 2005	Reported	Heparin 300 U/kg IV, maintain ACT > 300 s, reversal with protamine (1 mg per 100 U heparin)
Vijay, 2023	Reported	Heparin 300 U/kg IV, maintain ACT > 300 s, reversal with protamine (1 mg per 100 U heparin)
Wang, 2022	Reported	Heparin 300 U/kg IV, maintain ACT > 300 s, reversal with protamine (1 mg per 100 U heparin)
Wei, 2006	Reported	Heparinization with ACT > 250 s, reversal with protamine
Wei, 2006	Reported	Heparinization with ACT > 250 s, reversal with protamine
Weingarten, 2021	Reported	Heparin 300 U/kg IV, maintain ACT > 300 s, reversal with protamine (1 mg per 100 U heparin)
Yang, 2003	Reported	Heparin 300 U/kg IV, maintain ACT > 300 s, reversal with protamine (1 mg per 100 U heparin)

Table S12. Anticoagulation protocols in selected studies. ACT, Activated Clotting Time; UFH, Unfractionated Heparin; OPCAB, Off-Pump Coronary Artery Bypass.

S13) Grading of Recommendations Assessment, Development, and Evaluation (GRADE) assessment of the level of certainty of the evidence.

Question: Antifibrinolytic Agents compared to Control in patients undergoing off-pump CABG
Intervention: Use of antifibrinolytic agents (tranexamic acid, aprotinin and epsilon-aminocaproic)
Control: Placebo or no intervention

Certainty assessment							№ of patients		Effect		Certainty	Importance
№ of studies	Study design	Risk of bias	Inconsistency	Indirectness	Imprecision	Other considerations	Antifibrinolytic Agents	Control	Relative (95% CI)	Absolute (95% CI)		
**Overall Mortality (RCTs)**												
10	randomised trials	serious	not serious	serious[a]	not serious	none	20/711 (2.8%)	29/704 (4.1%)	**RR 0.72** (0.54 to 0.95)	**12 fewer per 1,000** (from 19 fewer to 2 fewer)	⨁⨁◯◯ Low[a]	
**Thromboembolic Events (RCTs)**												
17	randomised trials	not serious	not serious	serious[a]	not serious	none	20/570 (3.5%)	45/579 (7.8%)	**RR 0.55** (0.39 to 0.79)	**35 fewer per 1,000** (from 47 fewer to 16 fewer)	⨁⨁⨁◯ Moderate[a]	

CI: confidence interval; **RR:** risk ratio

Explanations

a. The evidence is rated down for indirectness because the included studies evaluated aprotinin, a drug that is either unavailable or tightly restricted in many jurisdictions. This means the results may not be generalizable to contexts where this intervention cannot be used.

S14) Search strategy

Pubmed

("Off-Pump Coronary Artery Bypass"[MeSH Terms] OR "off pump"[tiab] OR "off-pump"[tiab] OR "off pump coronary artery bypass"[tiab] OR "off-pump coronary artery bypass"[tiab] OR "off-pump CABG"[tiab] OR "off pump CABG"[tiab] OR "OPCAB"[tiab] OR "OP-CAB"[tiab]) AND ("Antifibrinolytic Agents"[MeSH Terms] OR "Tranexamic Acid"[MeSH Terms] OR "Aprotinin"[MeSH Terms] OR "tranexamic acid"[tiab] OR "antifibrinolytic"[tiab] OR "antifibrinolytics"[tiab] OR "cyklokapron"[tiab] OR "transamin"[tiab] OR "amcha"[tiab] OR "TXA"[tiab] OR "epsilon-aminocaproic acid"[tiab] OR "epsilon aminocaproic acid"[tiab] OR "aprotinin"[tiab])

Embase

('off pump coronary artery bypass'/exp OR 'off pump':ti,ab OR 'off-pump':ti,ab OR 'off-pump coronary artery bypass':ti,ab OR 'off pump coronary artery bypass':ti,ab OR 'off-pump cabg':ti,ab OR 'off pump cabg':ti,ab OR 'opcab':ti,ab OR 'op-cab':ti,ab) AND ('antifibrinolytic agent'/exp OR 'tranexamic acid'/exp OR 'epsilon aminocaproic acid'/exp OR 'aprotinin'/exp OR 'tranexamic acid':ti,ab OR 'antifibrinolytic':ti,ab OR 'antifibrinolytics':ti,ab OR 'cyklokapron':ti,ab OR 'transamin':ti,ab OR 'amcha':ti,ab OR 'txa':ti,ab OR 'epsilon-aminocaproic acid':ti,ab OR 'epsilon aminocaproic acid':ti,ab OR 'aprotinin':ti,ab)

Cochrane

("Off-Pump Coronary Artery Bypass" OR "off pump" OR "off-pump" OR "off pump coronary artery bypass" OR "off-pump coronary artery bypass" OR "off-pump CABG" OR "off pump CABG" OR "OPCAB" OR "OP-CAB") AND ("Antifibrinolytic Agents" OR "Tranexamic Acid" OR "Aminocaproic Acids" OR "Aprotinin" OR "tranexamic acid" OR "antifibrinolytic" OR "antifibrinolytics" OR "cyklokapron" OR "transamin" OR "amcha" OR "TXA" OR "epsilon-aminocaproic acid" OR "epsilon aminocaproic acid" OR "aprotinin")

S15) Risk of Bias

A) Risk of Bias - Rob 2

Risk of bias domains

Study	D1	D2	D3	D4	D5	Overall
Ahn et al., 2012	+	+	+	+	+	+
Bert et al.	+	+	+	+	+	+
Casati et al., 2001	+	+	+	+	+	+
Casati et al., 2004	+	+	+	+	+	+
Chakravarthy et al., 2012	-	+	-	+	+	-
Desai et al., 2009	+	+	+	+	+	+
Englberger et al., 2002	-	+	-	+	+	-
Grant et al., 2008	+	+	+	+	+	+
Hosseini et al., 2014	+	+	+	+	+	+
Kim et al., 2004	+	-	+	+	+	-
Mehr-Aein et al., 2007	+	+	+	+	+	+
Murphy et al., 2006	+	+	+	+	+	+
Nurözler et al., 2008	+	+	+	+	+	+
Poston et al., 2006	+	+	+	+	+	+
Taghaddomi et al., 2009	+	+	+	+	+	+
Vanek et al., 2005	+	+	-	+	+	-
Vijay et al., 2023	X	X	+	X	+	X
Wei et al., 2006 aprotinin	-	+	+	+	+	-
Wei et al., 2006 TXA	-	+	+	+	+	-
Yang et al.. 2004	-	+	+	+	+	-

Domains:
D1: Bias arising from the randomization process.
D2: Bias due to deviations from intended intervention.
D3: Bias due to missing outcome data.
D4: Bias in measurement of the outcome.
D5: Bias in selection of the reported result.

Judgement
X High
- Some concerns
+ Low

B) Risk of Bias - ROBINS-I

Risk of bias domains

Study	D1	D2	D3	D4	D5	D6	D7	Overall
Bittner, 2008	-	-	+	+	+	-	+	-
Durand, 2006	-	X	+	+	+	+	-	X
Hulde, 2019	-	-	-	+	+	-	+	-
Khandanga, 2020	-	-	+	+	+	-	+	-
Mouton, 2008	-	-	-	+	+	-	+	-
Wang et al. 2022	-	-	-	+	+	-	+	-
Weingarten et al. 2021	X	-	?	?	+	-	+	X

Domains:
D1: Bias due to confounding.
D2: Bias due to selection of participants.
D3: Bias in classification of interventions.
D4: Bias due to deviations from intended interventions.
D5: Bias due to missing data.
D6: Bias in measurement of outcomes.
D7: Bias in selection of the reported result.

Judgement
X Serious
- Moderate
+ Low
? No information
